# Age-specific reference values for carotid arterial stiffness estimated by ultrasonic wall tracking

**DOI:** 10.1038/s41371-019-0228-5

**Published:** 2019-08-21

**Authors:** Tokuhisa Uejima, Frank D. Dunstan, Eloisa Arbustini, Krystyna Łoboz-Grudzień, Alun D. Hughes, Scipione Carerj, Valentina Favalli, Francesco Antonini-Canterin, Olga Vriz, Dragos Vinereanu, Jose L. Zamorano, Bogdan A. Popescu, Arturo Evangelista, Patrizio Lancellotti, Georges Lefthériotis, Michaela Kozakova, Carlo Palombo, Alan G. Fraser, for the E-Tracking International Collaboration Group (ETIC)

**Affiliations:** 1 Wales Heart Research Institute, School of Medicine, Cardiff University, Cardiff, UK; 2 Department of Primary Care & Public Health, School of Medicine, Cardiff University, Cardiff, UK; 3 Centre for Inherited Cardiovascular Diseases, I.R.C.C.S. Foundation San Matteo Hospital, Pavia, Italy; 4 Department of Cardiology, T Marciniak Hospital, Wroclaw Medical University, Wroclaw, Poland; 5 Institute of Cardiovascular Science, University College London, London, UK; 6 Department of Internal Medicine and Pharmacology, University of Messina, Messina, Italy; 7 Azienda Ospedaliera S Maria degli Angeli, Pordenone, Italy; 8 Institute of Cardiology, Ospedale di San Daniele del Friuli, Udine, Italy; 9 University of Medicine and Pharmacy Carol Davila, University and Emergency Hospital, Bucharest, Romania; 10 Department of Cardiology, University Hospital Ramón y Cajal, Madrid, Spain; 11 Department of Cardiology, Euroecolab, Emergency Institute of Cardiovascular Diseases “Prof. Dr. C. C. Iliescu, Bucharest, Romania; 12 Department of Cardiology, Hospital General Universitari Vall d’Hebron, Barcelona, Spain; 13 GIGA Cardiovascular Science, Centre Hospitalier Universitaire de Liège Sart Tilman, Liège, Belgium; 14 Centre Hospitalier Universitaire de Nice, Unité de Médecine et Physiologie Vasculaire, Université Côte d’Azur, LP2M CNRS-7073, Nice, France; 15 Department of Surgical, Medical, Molecular Pathology and Critical Care Medicine, University of Pisa, Pisa, Italy; 16 Present Address: Rehabilitative Cardiology ORAS, Motta di Livenza, Treviso, Italy; 17 Present Address: Heart Centre, King Faisal Specialist Hospital, Riyadh, Saudi Arabia

**Keywords:** Risk factors, Ultrasonography

## Abstract

Interaction between arterial stiffness and hypertension plays an important role in the development of cardiovascular disease. Accordingly, assessment of arterial stiffness may provide a tool for estimating cardiovascular risk and monitoring therapy in hypertensive patients. Radiofrequency-based vascular ultrasound allows accurate noninvasive assessment of local mechanical properties of large arteries, but for its use in clinical practice, reference values according to age and sex are mandatory for each vascular site. To provide reference values for common carotid artery stiffness as assessed by an echo-tracking imaging system Hitachi-Aloka, we pooled measurements collected in 1847 healthy subjects aged 3–74 years (1008 males and 839 females) recruited in 14 European centers in the E-tracking International Collaboration (ETIC). Statistical models were developed to describe relationships of different stiffness indices with age and to calculate median values and *Z*-scores corresponding to ± 1 and ± 2 standard deviations. In our apparently healthy population, age accounted for 53% of variability in the elastic modulus (epsilon), 39% in arterial compliance, 47% in stiffness index (β), and 56% in local pulse wave velocity; on average, blood pressure accounted for a further 7.5% of variability. Dependence on age was not linear; changes in mean values increased at older ages, especially for epsilon and β. There was an interaction between age and gender for arterial compliance, which was higher in males. We present nomograms and a software that can be used for the automated calculation of *Z*-scores for local carotid stiffness in individual patients. These tools can be used to establish prognostic indicators or surrogate targets for treatment monitoring.

## Introduction

Arterial stiffening is a hallmark of ageing since it reflects changes in the mechanical properties of the arterial wall caused by progressive, age-related spatial disorganization and fragmentation of elastin and by accumulation and cross-linking of collagen [1]. The presence of hypertension accelerates age-dependent vascular stiffening, through an increase in distension pressure that promotes spatial redistribution of vascular smooth muscle cells (VSMCs) and remodeling of the extracellular matrix [2]. Hormones of the renin-angiotensin-aldosterone system may also be involved in the stiffening of the arterial wall through their impact on VSMCs, elastin and collagen, and through activation of inflammatory cytokines [3].

Arterial stiffness has been shown to predict cardiovascular (CV) morbidity and mortality in different populations [4, 5], including hypertensive patients [6, 7], and antihypertensive drugs have been shown to decrease arterial stiffness [8]. Therefore, the measurement of arterial stiffness in hypertensive subjects may be helpful both for the estimation of individual CV risk and for monitoring the efficacy of therapeutic intervention.

Several invasive and noninvasive methods have been proposed for the estimation of arterial stiffness. A recognized noninvasive standard is carotid-femoral pulse wave velocity (PWV) [9–11] that measures the speed of pulse wave propagation in the thoraco-abdominal aorta, that is, in a relatively long portion of the arterial tree that includes several segments with different mechanical characteristics due to different content of elastin, collagen and VSMCs [12]. Since age, high blood pressure (BP) and antihypertensive treatment may influence various components of the arterial wall in a different way, the assessment of local mechanical properties in one or more specific segments may be of interest. Indeed, it has been shown in hypertensive patients that the impact of age on arterial stiffness differs between the aorta and the carotid arteries [13], that aortic and carotid stiffness are differently associated with target organ damage [14], that hypertension-induced arterial wall hypertrophy is associated with a decreased distensibility in carotid but not in radial arteries [15] and that the arterial effect of antihypertensive drugs administered at a given dose and period of time may differ according to the arterial site [8, 16].

The assessment of local carotid stiffness is of particular interest as stiffening of the carotid arteries reduces their cushioning function and increases pressure and flow pulsatility in the cerebral circulation. These haemodynamic alterations are believed to increase the risk of stroke, cognitive impairment, and dementia [17, 18]. However, the use of carotid stiffness in individual risk assessment and in monitoring of treatment is limited by the lack of reference values according to age, sex, and BP.

Measurement of local carotid stiffness is based on the evaluation of pressure–diameter relationships, and ultrasound is usually used to measure the changes in diameter. The video signal of a routine ultrasound examination has inadequate temporal and spatial resolution to follow the rapid displacement of the arterial wall during the cardiac cycle, so high-resolution wall tracking systems analyzing the raw radiofrequency signals have been developed [19]. A study comparing two commercially available wall tracking systems indicates that the values of carotid distension are not inter-changeable [20], and that reference data are needed for each system [21]. To establish age-specific reference values for carotid stiffness indices as obtained with the echo-tracking system E-track^®^ of Hitachi-Aloka and to evaluate their relationships with gender, BP, heart rate, and body size, we set-up the ETIC that generated a large database of measurements of carotid arterial stiffness in healthy subjects in 14 centers across Europe.

## Materials/subjects and methods

The ETIC database pooled data from healthy subjects aged 3–74 years who had ultrasound scans of the common carotid artery (CCA) for the assessment of arterial stiffness, using the E-track^®^ technique (Hitachi-Aloka; Tokyo, Japan). Data were collected in 14 centers across Europe (online Supplement). The protocol of the study followed the principles of the Declaration of Helsinki and each participating center had obtained approval from its local research ethics committee and informed consent from each subject or, in case of minors, from the parent. To maintain confidentiality, data were transferred to a secure website and anonymized by allocating each subject a new number. Data recorded at the time of study included age, gender, body height, and weight, body mass index (BMI), heart rate, and systolic and diastolic BP.

Sample size calculation [22] was performed using pilot data from a single center (Pisa) that included 166 apparently healthy subjects (82 men, age 15–68 years) who had the mean value of local carotid PWV = 5.45 ± 1.33 m/s and its age-dependent increase = 0.089 m/s per year. Applying the calculation of sample size with an alpha error = 0.05 and power = 0.80, 1750 subjects resulted adequate with two-sided test and 1385 subjects with one-sided test.

Subjects were excluded if they had any of the following: (1) systolic BP > 140 or diastolic BP > 90 mmHg, (2) treated hypertension, (3) type 1 or 2 diabetes mellitus, (4) treated hypercholesterolemia, (5) symptomatic or confirmed coronary artery disease, (6) clinical cerebrovascular disease, (7) carotid arterial plaque, (8) severe peripheral vascular disease, (9) hypertrophic or dilated cardiomyopathy, (10) congestive heart failure or LV EF < 50%, (11) heart valve disease, (12) previous cardiac surgery, (13) congenital heart disease, (14) systemic diseases such as cancers, endocrine, inflammatory and autoimmune diseases, (15) current smoking, and (16) any regular drug treatment.

### Measurements of carotid stiffness

Subjects were studied after resting supine for >10 min. The right CCA was scanned using a Hitachi-Aloka SSD5500 or a Prosound α10 ultrasound system with a 10 MHz linear array vascular transducer. Where adequate imaging of the right CCA was impossible, images were acquired from the left CCA (in <10%). Change in diameter was measured as the difference between displacement waveforms of the anterior and posterior walls, with cursors set manually about one centimeter proximal to the carotid sinus, for automated tracking of the media-adventitia boundaries in the arterial wall at a sampling frequency of 2 kHz. The diameter waveform was calibrated for pressure using simultaneously acquired brachial systolic and diastolic BP as previously reported [23]. Automated oscillometric devices were used (Omron 705 cp, Kyoto, Japan).

Peterson pressure–strain elastic modulus (epsilon—Ep), beta stiffness index (β), arterial compliance (AC), and local carotid PWV were calculated as described in the online Supplement. We have reported the reproducibility of these measurements elsewhere [24].

### Measurement of central pressure

In 76 subjects (46 males, age range 16–64 years) investigated in one center (Pisa), carotid applanation tonometry (PulsePen; Diatecne, Milan) was performed simultaneously to the acquisition of distension curves by echo-tracking on the contralateral CCA [25]. Local carotid pressure was obtained from alternative calibration of the pressure waveforms, and stiffness indices were recalculated after replacing brachial pressure with local carotid (central) pressure.

### Statistical analysis

The data were inspected for aberrant values, and summary statistics were calculated and presented as mean and standard deviation. Comparisons were made to ensure there were no systematic variations between centers. The Altman’s method was used to derive age-related centiles for the stiffness parameters [26].

The data were transformed, using the Box–Cox procedure to select an appropriate transformation, to produce approximately normally distributed (Gaussian) data [27]. The dependence of the mean on age was modeled by a cubic function, fitted by linear regression, with a linear function of age for the standard deviation based on regression modeling of the absolute residuals. For some variables there was evidence of different relationships with age in subjects <18 years, compared with adults, so segmented regression was used to fit different models in these two broad age groups; this ensured continuity in predicted values for ages below and above 18 years. Age-dependent centiles were calculated based on these Gaussian distributions and the transformations were inverted to produce centiles for the original variables. *Z*-scores of ±2, ±1, and 0 were calculated and plotted, corresponding to 2.5th, 16th, 50th, 84th, and 97.5th centiles (encompassing mean values ±1 or ±2 standard deviations).

Interactions between age and gender were tested in all subjects by general linear modeling on the transformed data, with age treated as a categorical variable using the bands shown in Table 1.

**Table 1:** Summary of demographic data

	Numbers of subjects		Resting heart rate (bpm)		Systolic blood pressure (mmHg)		Diastolic blood pressure (mmHg)		Pulse pressure (mmHg)		Body mass index (kg/m^2^)	
Age range (years)	Male	Female	Male	Female	Male	Female	Male	Female	Male	Female	Male	Female
3–10	93	71	77.3 (12.2)	82.1 (13.3)	103.6 (8.8)	103.3 (10.1)	63.8 (8.0)	64.3 (8.2)	39.9 (7.7)	39.0 (6.1)	17.4 (3.6)	16.9 (3.0)
11–17	166	101	64.1 (10.7)	70.0 (10.6)	114.9 (8.7)	109.5 (9.9)	67.8 (8.8)	66.8 (8.2)	47.1 (9.5)	42.7 (8.5)	20.4 (3.0)	19.6 (3.4)
18–29	269	183	63.3 (10.8)	68.2 (11.3)	120.0 (9.5)	110.9 (10.3)	71.6 (8.2)	69.4 (7.7)	48.4 (9.1)	41.5 (8.5)	23.2 (3.0)	21.5 (2.9)
30–39	172	176	63.3 (10.3)	67.4 (11.1)	120.8 (9.4)	113.7 (10.8)	74.2 (7.9)	71.3 (8.7)	46.6 (8.7)	42.4 (8.1)	25.2 (3.7)	22.8 (3.8)
40–49	129	139	62.6 (12.2)	68.0 (10.5)	122.5 (10.5)	117.0 (11.2)	77.5 (8.0)	73.9 (8.3)	45.0 (8.1)	43.0 (8.6)	25.7 (3.6)	23.8 (4.0)
50–59	83	111	63.1 (10.2)	65.7 (9.5)	122.3 (10.5)	123.3 (11.5)	77.4 (7.3)	76.1 (6.9)	44.9 (8.8)	47.2 (8.6)	26.7 (2.8)	24.2 (3.7)
60–74	96	57	63.2 (10.4)	63.5 (10.8)	125.5 (8.5)	122.4 (12.3)	77.4 (6.4)	72.7 (8.0)	47.7 (7.4)	49.6 (10.3)	28.6 (8.2)	26.1 (5.1)

Data reported as mean value (s.d.) for all subjects within each decade (or different age range as shown)

## Results

We studied 1847 healthy subjects (1008 males and 839 females) aged 3–74 years (Table 1); the number per center ranged from 37 to 927. There were no significant differences in arterial function between centers.

The mean heart rate was higher in younger children compared with other age–gender groups, and it was also higher in women than in men (*p* < 0.001). The heart rate decreased with age, with a greater rate of decrease in women. Systolic BP and pulse pressure were higher in men (*p* < 0.001) but they increased with age more in women so that the difference between genders narrowed (interaction, *p* < 0.001). Diastolic BP increased with age (*p* < 0.001) with a higher rate of increase in men. BMI was higher, and it increased more with age, in men (*p* < 0.001).

### Indices and determinants of local carotid stiffness

**Fig. 1:**
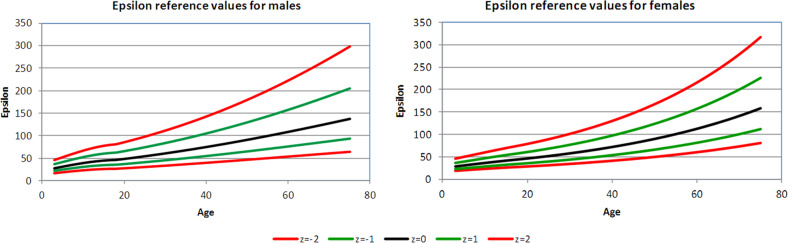
Carotid Ep displayed as median value, ±*Z*-scores, separately for men and women

The mean values of Ep, β, AC, and local PWV are given in Table 2, by age bands and gender. Ep, β, and PWV were modeled after log transformations; AC was transformed by raising the values to the power ¼ (online Supplement). Age-related means and standard deviations were estimated as described in the methods and used to calculate centiles of the transformed variables; the transformations were inverted to derive centiles for the original variables. The outcome is illustrated in Fig. 1S, which shows the individual data for one measurement (Ep) with the results of the models superimposed in order to indicate the estimated median values and *Z*-scores according to the age and gender.

**Table 2:** Mean values of indices of local carotid arterial stiffness by age bands and gender

	Ep (kPa)		Beta stiffness index		Arterial compliance (mm^2^/kPa)		Pulse wave velocity (m/s)	
Age range (years)	Male	Female	Male	Female	Male	Female	Male	Female
Children and adolescents								
3–10	37.7 (12.6)	34.9 (9.5)	3.46 (1.11)	3.21 (0.85)	1.65 (0.50)	1.54 (0.46)	3.66 (0.58)	3.58 (0.46)
11–17	42.6 (12.0)	43.3 (13.2)	3.76 (1.05)	3.76 (1.14)	1.51 (0.40)	1.47 (0.40)	3.96 (0.50)	3.94 (0.54)
Adults								
18–29	54.2 (15.8)	52.2 (15.4)	4.33 (1.27)	4.44 (1.28)	1.36 (0.38)	1.23 (0.38)	4.40 (0.75)	4.30 (0.69)
30–39	68.5 (22.3)	66.2 (19.3)	5.38 (1.73)	5.49 (1.59)	1.11 (0.31)	0.95 (0.29)	4.97 (0.74)	4.91 (0.67)
40–49	84.8 (29.3)	83.4 (24.4)	6.46 (2.14)	6.64 (1.82)	1.01 (0.34)	0.79 (0.24)	5.49 (1.00)	5.52 (0.79)
50–59	102.0 (38.0)	96.4 (32.1)	7.61 (2.83)	7.34 (2.20)	0.90 (0.32)	0.76 (0.24)	6.14 (1.32)	5.92 (1.11)
60–74	121.6 (48.5)	130.2 (48.0)	9.15 (3.55)	10.30 (3.79)	0.82 (0.31)	0.70 (0.29)	6.62 (1.29)	6.80 (1.27)

Data reported as mean value (s.d.) for all subjects within each decade (or different age range as shown). For number of subjects in each cell, see Table 1.

**Fig. 2:**
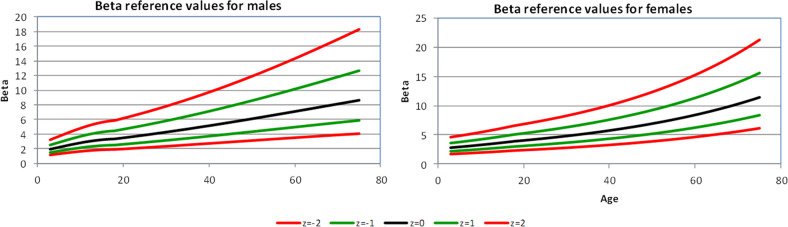
Carotid β displayed as median value, ±*Z-*scores, separately for men and women

**Fig. 3:**
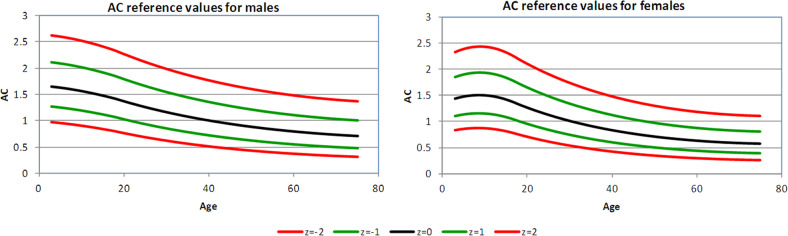
Carotid AC displayed as median value, ±*Z-*scores, separately for men and women

**Fig. 4:**
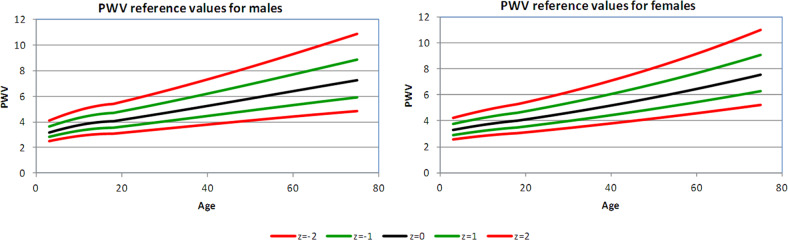
Carotid PWV displayed as median value, ±*Z-*scores, separately for men and women

Figures 1–4 show the 2.5th, 16th, 50th, 84th, and 97.5th centiles for all the parameters, presented as median values and *Z*-scores; these relate to ±1 and ±2 standard deviations from the mean, of the transformed, approximately normally distributed, variables. The percentages of values lying between the estimated centiles were calculated as a check and were always close to the nominal values. Supplementary Tables S1–S4 (online Supplement) give values corresponding to *Z*-scores by age for each variable, separately in male and female subjects. An electronic calculator (Excel spreadsheet) that can be used to calculate *Z*-scores for individual measurements is also available at the online Supplement.

In subjects undergoing carotid applanation tonometry, the values of all stiffness indices were strongly correlated with the corresponding ones recalculated after replacing brachial BP with local carotid BP [Spearman’s correlation coefficient for Ep 0.98, for β 0.98, for AC 0.97, and for PWV 0.99 (all, *p* < 0.0001)].

#### Pressure–strain (Peterson’s) elastic modulus

Ep increased from the third to the seventh decade (aged 18–29 to 60–74 years) by a factor of 2.2 in men compared with 2.5 in women (Table 2). There was no significant overall difference between the genders (*p* = 0.31) and no significant interaction between the age and gender (*p* = 0.50).

#### Beta stiffness index

β increased over the age range by a factor of 2.1 in men and 2.3 in women (Table 2). There was no significant difference between genders (*p* = 0.15) nor any significant interaction between age and gender (*p* = 0.22). There was a small positive association between β and mean BP, but after adjusting for age and gender, the association changed to a small negative one. The percentage of variation explained was 55.7% before and 56% after adding mean BP to a model including age and gender, so that the BP effect is negligible (regression coefficient −0.0026). In this study, beta stiffness index was thus independent of mean BP.

#### Arterial compliance

The relative reductions in AC (i.e. the inverse of stiffness) between ages 18–29 and 60–74 years were 40% for men and 43% for women (Table 2). AC was higher in men (*p* < 0.001); the gap between males and females gradually widened with increasing age (interaction, *p* = 0.018). The diameter of the CCA was larger in men than in women, by an average of 0.56 mm (in 935 men, 7.2 ± 0.8 mm; compared with 756 women, 6.7 ± 0.8 mm; *p* < 0.0001).

#### Pulse wave velocity

The relative increase in local PWV over the observed age span was 1.5 in both men and women; there was no difference between genders.

For all the measured indices of local arterial function, age was the most important predictor, accounting for 39–56% of variation observed (based on *R*^2^ from multivariate analyses; mean 48.8%; Table 3). On average, adding BP to regression models accounted for a further 7.5% of variation. Adding heart rate, BMI, and gender, accounted for another 3.7%.

**Table 3:** Percentage of variation attributable in different models

	Age	Age and BP^a^	Age and BP^a^ and other factors
Ep (kPa)	53.4%	62.6%	65.5%
Beta index	46.9%	54.5%	57.1%
Arterial compliance (mm^2^/kPa)	39.2%	46.8%	53.7%
Pulse wave velocity (m/s)	55.8%	61.3%	63.5%

^a^For Ep, beta, and arterial compliance, calculated using arterial pulse pressure; for pulse wave velocity, calculated using systolic BP

## Discussion

This study presents age-dependent reference values for carotid stiffness indices obtained in a large sample of apparently healthy subjects who were examined using the Hitachi-Aloka ultrasound wall-tracking system. As expected, Ep, β, and local PWV increased with age; for Ep and β the increase was slightly accelerated in later decades, whereas age-related increase in local PWV was almost linear. AC showed similar changes in an opposite direction. Reference ranges gradually widened with age, as also reported for carotid-femoral PWV [11]. In general, the pattern of age-related arterial stiffening was similar for men and women. In comparison with the impact of ageing, the variation in indices of carotid arterial stiffness with arterial BP was small.

### Defining normality

There is no consensus on how to identify a “normal” population for reference when interpreting CV imaging. There is a continuum from perfect health, through asymptomatic subjects with CV risk factors, to patients with early disease. We considered subjects with no history of overt CV disease and without diabetes, high BP, or dyslipidaemia, to be appropriate for the reference database that we used to develop an algorithm to assess physiologic vascular ageing. We recognize that this approach to defining normality becomes less representative of the general population at older ages. We report *Z*-scores to avoid overdiagnosis and to provide simple diagnostic tools developed from the statistical analysis of many subjects. Some authors reported a different rate of change of arterial stiffness during childhood and adolescence compared with adult life [28]. We allowed for such effects during the modeling, while constraining the results to ensure continuity below and above the age of 18 years. The segmented regression analysis explains the minor inflections observed in some nomograms at age 18, and perhaps also the unusual pattern of change observed in AC in young female subjects.

### Device-specific reference ranges

As diagnostic imaging becomes more sophisticated, the algorithms for processing radiofrequency signals generated from ultrasound transducers become more precise but also more complex. Thus, measurements obtained using one system cannot be assumed to be equivalent to those obtained using a similar system from a different manufacturer [20]. Unless a common standard shared by different manufacturers will be developed and applied, normal values should be reported for each diagnostic system [21].

### Local PWV

PWV is a simple, reproducible index of arterial stiffness that is directly related to the elastic property of arterial walls [2]. Carotid-femoral measurement averages PWV over several arterial segments with diverse elastic properties, whereas the one-point method reported in our study determines PWV at a single region of interest. This may be useful clinically since different segments of the arterial tree are affected differently by ageing, hypertension and, treatment [13–16].

Elastin is a major constituent of the thoracic aorta while collagen predominates outside the thorax. Ageing, with derangement of elastin fibers and increase in collagen content, is likely to affect the thoracic aorta more. Whereas aortic PWV almost doubles between the third and seventh decades [11], the age-related increase in local carotid PWV in our study is lower, being 42% in men and 51% in women (see Supplementary Tables 4a and 4b). A study comparing carotid-femoral and local carotid PWV in the same population reported that carotid-femoral PWV showed a steeper age-dependent increase in subjects older than 50 years of age as compared with carotid PWV [29]. A more prominent age-dependent increase in carotid-femoral PWV in normotensive individuals over 50 years of age was also described in the Anglo-Cardiff Collaborative Trial [30]. These observations further confirm the need for age-adjusted reference PWV values in different vascular territories.

In the ETIC study, the age-related increase in local carotid PWV did not differ significantly between genders, which agrees with data on carotid-femoral PWV that reported either no differences between sexes [29, 30] or statistically significant but negligible differences (0.1 m/s higher in men than in women) [11].

### Elastic modulus, beta index, and AC

The pressure–strain elastic modulus, Ep, was introduced by Peterson to describe the mechanical property of arterial walls [31]. The pressure–strain relation is curvilinear and related to BP; to reduce this effect, Hayashi proposed the stiffness index β, which incorporates a simple exponential correction [32]. In the ETIC study, age-dependent increases of Ep and β were slightly accelerated in later decades, and there were no differences between the genders. As expected AC decreased with age and there was an interaction between age and gender; AC was higher in males, with the gap between genders widening with increasing age. The difference between genders can in part be explained by the smaller diameter of the carotid artery in women.

### Carotid stiffness and CV risk

Several clinical studies have demonstrated the associations of carotid stiffness measures with CV events. In the Atherosclerosis Risk in Communities (ARIC) study of >10,000 subjects followed for 13.8 years, carotid distensibility and Ep had an independent predictive value for incident stroke but not for coronary heart disease, after adjusting for known risk factors [33]. An independent association between carotid stiffness and stroke was confirmed also by meta-analysis including 22,470 subjects [17]. Finally, in the Hoorn study, carotid stiffness predicted CV events and all-cause mortality at 7 years, with 50% increased risk in those with the stiffest arteries [34].

### Limitations

First, we used brachial BP as a substitute for carotid BP. In younger subjects, systolic BP and pulse pressure are generally higher in the brachial artery than the central aorta (pulse pressure amplification), while in older subjects brachial and central BPs are more similar. Rescaling and calibrating the local arterial distension waveform generated by wall tracking provides an accurate estimate of local carotid pressure [25], yet the algorithm used in our study and made available by the manufacturer does not use extrapolated central arterial pressures to compute the stiffness indices. Therefore, carotid Ep, β, and PWV could have been overestimated especially in younger subjects by using brachial rather than carotid pressures. However, results obtained in the subset of subjects undergoing carotid applanation tonometry simultaneously with arterial wall tracking indicate that there was no significant bias.

Second, we have no data on plasma glucose and lipids, which were shown to be independent predictors of carotid-femoral PWV in different studies [13, 29, 35]. However, in a population similar to that of this study, i.e. free of CV disease, diabetes, antihypertensive and lipid-lowering treatment, carotid-femoral but not local carotid PWV was related to plasma lipids and glucose [29]. Third, we did not consider ethnicity, which might also influence arterial stiffness [36]. Finally, ETIC was a cross-sectional study that was not designed to provide follow-up data.

## Conclusions

New diagnostic imaging tests are often implemented before their normal ranges have been established, which makes integration into clinical practice difficult. Reference ranges are required for each new test of vascular function obtained at each vascular site with each diagnostic system. The ETIC study has established reference ranges for measurements of local arterial stiffness in the CCA with the ultrasound wall-tracking system Hitachi-Aloka. The online calculator of *Z*-scores according to the age and gender can be used to interpret tests and to track changes in individual hypertensive subjects. The clinical utility of using these indices as surrogate end-points can now be studied in trials.

### Summary table

#### What is known about topic

- Local carotid stiffness has been shown to predict incident stroke, cardiovascular events, and all-cause mortality.
- Radiofrequency-based vascular ultrasound allows accurate noninvasive assessment of local carotid artery stiffness, yet, reference values according to age and sex are mandatory for its use in clinical practice.

#### What this study adds

- The present study established, in a population of 1.847 apparently healthy subjects (aged 3–74 years), reference ranges for indices of local carotid stiffness as obtained by the ultrasound wall-tracking system Hitachi-Aloka.
- The study provides nomograms and a software, which can be used for the automated calculation of *Z*-scores for local carotid stiffness, according to age and gender. These tools can be used to establish prognostic indicators or surrogate targets for treatment monitoring.

## Supplementary information

Supplemental Material

Supplemental Figure

ETIC Calculator of z scores
