# Correction: Age-specific reference values for carotid arterial stiffness estimated by ultrasonic wall tracking

**DOI:** 10.1038/s41371-020-0304-x

**Published:** 2020-01-30

**Authors:** Tokuhisa Uejima, Frank D. Dunstan, Eloisa Arbustini, Krystyna Łoboz-Grudzień, Alun D. Hughes, Scipione Carerj, Valentina Favalli, Francesco Antonini-Canterin, Olga Vriz, Dragos Vinereanu, Jose L. Zamorano, Bogdan A. Popescu, Arturo Evangelista, Patrizio Lancellotti, Georges Lefthériotis, Michaela Kozakova, Carlo Palombo, Alan G. Fraser, for the E-Tracking International Collaboration Group (ETIC)

**Affiliations:** 1 Wales Heart Research Institute, School of Medicine, Cardiff University, Cardiff, UK; 2 Department of Primary Care & Public Health, School of Medicine, Cardiff University, Cardiff, UK; 3 Centre for Inherited Cardiovascular Diseases, I.R.C.C.S. Foundation San Matteo Hospital, Pavia, Italy; 4 Department of Cardiology, T Marciniak Hospital, Wroclaw Medical University, Wroclaw, Poland; 5 Institute of Cardiovascular Science, University College London, London, UK; 6 Department of Internal Medicine and Pharmacology, University of Messina, Messina, Italy; 7 Azienda Ospedaliera S Maria degli Angeli, Pordenone, Italy; 8 Institute of Cardiology, Ospedale di San Daniele del Friuli, Udine, Italy; 9 University of Medicine and Pharmacy Carol Davila, University and Emergency Hospital, Bucharest, Romania; 10 Department of Cardiology, University Hospital Ramón y Cajal, Madrid, Spain; 11 Department of Cardiology, Euroecolab, Emergency Institute of Cardiovascular Diseases “Prof. Dr. C. C. Iliescu, Bucharest, Romania; 12 Department of Cardiology, Hospital General Universitari Vall d’Hebron, Barcelona, Spain; 13 GIGA Cardiovascular Science, Centre Hospitalier Universitaire de Liège Sart Tilman, Liège, Belgium; 14 Centre Hospitalier Universitaire de Nice, Unité de Médecine et Physiologie Vasculaire, Université Côte d’Azur, LP2M CNRS-, 7073 Nice, France; 15 Department of Surgical, Medical, Molecular Pathology and Critical Care Medicine, University of Pisa, Pisa, Italy; 16 Present Address: Rehabilitative Cardiology ORAS, Motta di Livenza, Treviso, Italy; 17 Present Address: Heart Centre, King Faisal Specialist Hospital, Riyadh, Saudi Arabia

**Keywords:** Risk factors, Ultrasonography

**Correction to: Journal of Human Hypertension**

10.1038/s41371-019-0228-5

This aticle was originally published under Nature Research’s License to publish, but has now been made available under a [CC BY 4.0] license. The PDF and HTML versions of the article have been modified accordingly.

