# Supplementary material for: Age-specific reference values for carotid arterial stiffness estimated by ultrasonic wall tracking
Source: J Hum Hypertens. 2019 Aug 21;34(3):214–22. doi: 10.1038/s41371-019-0228-5 (PMC8076029; doi:10.1038/s41371-019-0228-5)
Supplement: Supplementary file 1 — Supplemental Material [file 41371_2019_228_MOESM1_ESM.docx]

**Summary:**

Supplementary Material provides information on participating centers, includes formulas used for calculation of indices of carotid stiffness and Z-scores as well as tables giving values corresponding to Z scores by age for each variable, separately in men and women. An electronic calculator (Excel spreadsheet) that can be used to calculate Z-scores for individual measurements is also included.

**Participating Centers**

| **ETIC Collaborators** | **Centres** | **Affiliation** |
| --- | --- | --- |
| Professor Eloisa Arbustini  Eng. Valentina Favalli  Dr Alessandra Serio  Dr Fabiana I Gambarin  Dr Michele Pasotti | Pavia, Italy | Centre for Inherited Cardiovascular Diseases, IRCCS Policlinico San Matteo |
| Professor Krystyna Łoboz-Grudzień  Dr Joanna Jaroch  Dr Zbigniew Bociąga | Wroclaw, Poland | Department of Cardiology, T Marciniak Hospital, Wroclaw Medical University |
| Dr Francesco Antonini-Canterin | Pordenone, Italy | Unità Operativa di Cardiologia, ARC, Azienda Ospedaliera S Maria degli Angeli |
| Dr Olga Vriz | Udine, Italy | Institute of Cardiology, San Daniele Hospital |
| Professor Alan Fraser  Dr Tokuhisa Uejima  Professor Frank Dunstan*  Dr Ye Kyaw | Cardiff, U.K. | Wales Heart Research Institute, and *Department of Primary Care & Public Health, School of Medicine, Cardiff University |
| Professor Carlo Palombo  Dr. Carmela Morizzo*  Dr. Michaela Kozàkovà* | Pisa, Italy | Department of Surgical, Medical, Molecular Pathology and Critical Medicine, and *Department of Clinical and Experimental Medicine; University of Pisa |
| Professor Scipione Carerj  Dr Concetta Zito | Messina, Italy | Department of Internal Medicine and Pharmacology, University of Messina |
| Professor Alun Hughes  Dr Chloe Park | London, U.K. | National Heart and Lung Institute, Imperial College, and Institute of Cardiovascular Science, University College London |
| Professor Dragos Vinereanu  Dr Stefania Magda  Dr Maria Florescu  Dr Andrea Olivia Ciobanu | Bucharest, Romania | Department of Cardiology, University of Medicine and Pharmacy Carol Davila |
| Professor Jose Zamorano | Madrid, Spain | University Hospital Ramón y Cajal |
| Dr Bogdan A Popescu  Dr Andreea Călin  Dr Monica Roşca  Professor Carmen Ginghină | Bucharest, Romania | Department of Cardiology, "Prof. Dr. C. C. Iliescu" Institute of Cardiovascular Diseases, and University of Medicine and Pharmacy Carol Davila |
| Professor Arturo Evangelista | Barcelona, Spain | Department of Cardiology, Hospital General Universitari Vall d’Hebron |
| Professor Patrizio Lancellotti | Liège, Belgium | Department of Cardiology, Centre Hospitalier Universitaire de Liège |
| Professor Georges Lefthériotis | Nice, France | Department of Physiology, Centre Hospitalier Universitaire |

**Calculation of carotid stiffness indices by E-track®**

Peterson pressure-strain elastic modulus (epsilon – Ep), beta stiffness index (β), arterial compliance (AC), and local PWV were calculated as described below:

Ep = (Ps – Pd) / [(Ds – Dd) / Dd] (kPa)

β = ln (Ps / Pd) / [(Ds – Dd) / Dd] (dimensionless)

AC = (Areas Aread) / (Ps – Pd) (mm^2^/kPa)

PWV = (βPs / 2ρ)^1/2^ (m/s),

where Ps and Pd are systolic and diastolic blood pressure; Ds and Dd are maximum and minimum diameters of the artery measured by the wall tracking technique; Areas and Aread are maximum and minimum areas as calculated from diameters and ρ the density of blood (1050 Kg/m^3^). These parameters were analyzed using a dedicated software package (DAS-RS1, Hitachi-Aloka, Tokyo, Japan). The E-tracking system tracks radiofrequency signals before post-processing, and uses the same software to analyze arterial stiffness indices irrespective of the machine used to acquire the images.

Local PWV was calculated using the one-point method, from measurements of pressure and diameter obtained at one site in the common carotid artery. This method exploits the Bramwell-Hill equation [1], and the derivation of the formula [2] is summarized below:

Based on the formula for stiffness index β, we can assume the following relation between pressure and diameter of the artery:

β = ln(P(t) / P(ed)) / [(D(t) – D(ed)) / D(ed)], (1)

where P(t) is instantaneous pressure at a given time t, (ed) represents end-diastole, and D(t) is instantaneous diameter of the artery at a given time t.

Differentiating the above formula with respect to D(t) gives:

dP(t) / dD(t) = β P(t) / D(ed). (2)

Since the cross-section of the carotid artery is assumed to be circular, the following equation can be derived:

dP(t) / dA(t) = (β P(t) / 2) /(D(t) D(ed) / 4), (3)

where A(t) is instantaneous cross-sectional area of the artery at a given time t. The carotid artery shows a typical diameter change less than 10% during a cardiac cycle under physiological conditions. Given that D(ed) is approximated by D(t) in the above equation, then

dP(t) / dA(t) = β P(t) / 2A(t). (4)

The Bramwell-Hill equation relates PWV to distensibility of the arterial wall as below:

PWV^2^ = V dP(t) / ρ dV(t), (5)

where V is the volume of the arterial segment. Since elongation of the carotid artery during systole is negligible, V can be replaced by A:

PWV^2^ = (A(t) /c) (dP(t) / dA(t)). (6)

Substituting equation (4) into equation (6) gives the final formula used to define local PWV:

PWV = (β P(t) / 2ρ)^1/2^ (7)

The intra-observer and inter-observer coefficients of variation in the ETIC study were 4.9 and 7.0% for Ep; 3.8 and 7.2% for β; and 5.5 and 6.3% for PWV.

**References**

1. Bramwell JC, Hill AV. The velocity of the pulse wave in man. Proc R Soc Lond 1922;93:298–306.
2. Sugawara M, Niki K, Furuhata H, Ohnishi S, Suzuki S, Relationship between the pressure and diameter of the carotid artery in humans. *Heart Vessels* 2000; 15: 49-51.

**Statistical modeling – calculation of Z scores**

The table shows the parameters that were derived from the modeling and then used to calculate Z-scores for each index, using the formulae given below:

|  | | **a** | **b** | **c** | **d** | **e** | **f** | **g** |
| --- | --- | --- | --- | --- | --- | --- | --- | --- |
| **Ep** | Males | 3.154 | 0.0208 | -0.000122 | -0.0981 | -0.00214 | 0.252 | 0.0016 |
|  | Females | 4.001 | 0.021 | -0.000001 | -0.0187 | -0.00055 | 0.228 | 0.00076 |
| **β** | Males | 0.692 | 0.0186 | -0.0000853 | -0.0843 | -0.002 | 0.252 | 0.00136 |
|  | Females | 1.744 | 0.018 | 0.0000388 | 0.0045 | 0.000033 | 0.22 | 0.0016 |
| **AC** | Males | 101.604 | -0.372 | 0.00326 | -0.172 | -0.0088 | 6.17 | 0.0146 |
|  | Females | 84.006 | -0.453 | 0.0064 | -1.585 | -0.0451 | 5.83 | 0.0031 |
| **PWV** | Males | 1.058 | 0.011 | -0.000073 | -0.05 | -0.001 | 0.128 | 0.00092 |
|  | Females | 1.432 | 0.011 | -0.0000218 | -0.014 | -0.00031 | 0.117 | 0.0005 |

The general method for Ep, β and local PWV:

Calculate, where Age 1 = age – 35, if subject’s age ≤17 years, and Age 1 = – 17, if age ≥18 years:

Mean = a + [b × (age-35)] + [c × (age-35)^2^] + (d × age1) + (e × age1^2^), *and*

SD = 1.2533 × [f+g×(age-35)]

Then, if the variable of interest (Ep, β, pulse wave velocity) is denoted by Y, calculate

Z = (ln(Y) – mean)/SD

where ln(Y) denotes the natural logarithm of Y.

Then Z is the Z-score in the log domain and is approximately normally distributed.

For arterial compliance, calculate the mean and standard deviation as above, but then take

Z = (100×ac^0.25^ – mean)/SD

The results of the application of these models to each of the measurements of arterial function, are given in the next Tables (S1-S4).

**Supplemental Figure Legend**

Figure S1

Data from individual subjects (open circles) with superimposed results from the modelling of changes

in Ep with age – showing median value (green), ±1 standard deviation (blue), and ±2 standard

deviations (red).

**Supplemental Tables**

**Table S1a Peterson pressure-strain elastic modulus (Ep; kPa) values for males corresponding to specified z-scores**

|  | **z-score** | | | | | | | | | | |
| --- | --- | --- | --- | --- | --- | --- | --- | --- | --- | --- | --- |
| Age | **-2.5** | **-2** | **-1.5** | **-1** | **-0.5** | **0** | **0.5** | **1** | **1.5** | **2** | **2.5** |
| 10 | 20.26 | 23.14 | 26.42 | 30.18 | 34.46 | 39.36 | 44.95 | 51.34 | 58.63 | 66.96 | 76.48 |
| 20 | 23.32 | 26.90 | 31.04 | 35.80 | 41.30 | 47.65 | 54.96 | 63.40 | 73.14 | 84.38 | 97.34 |
| 30 | 27.99 | 32.61 | 38.00 | 44.27 | 51.59 | 60.11 | 70.04 | 81.61 | 95.10 | 110.81 | 129.11 |
| 40 | 32.77 | 38.57 | 45.40 | 53.43 | 62.88 | 74.01 | 87.11 | 102.52 | 120.66 | 142.01 | 167.14 |
| 50 | 37.45 | 44.52 | 52.93 | 62.92 | 74.80 | 88.92 | 105.71 | 125.68 | 149.40 | 177.61 | 211.15 |
| 60 | 41.77 | 50.15 | 60.22 | 72.31 | 86.83 | 104.27 | 125.21 | 150.35 | 180.54 | 216.79 | 260.32 |
| 70 | 45.46 | 55.13 | 66.87 | 81.11 | 98.37 | 119.32 | 144.72 | 175.53 | 212.90 | 258.22 | 313.20 |

**Table S1b Peterson pressure-strain elastic modulus (Ep; kPa) values for females corresponding to specified z-scores**

|  | **z-score** | | | | | | | | | | |
| --- | --- | --- | --- | --- | --- | --- | --- | --- | --- | --- | --- |
| Age | **-2.5** | **-2** | **-1.5** | **-1** | **-0.5** | **0** | **0.5** | **1** | **1.5** | **2** | **2.5** |
| 10 | 19.00 | 21.66 | 24.69 | 28.14 | 32.08 | 36.57 | 41.68 | 47.52 | 54.16 | 61.74 | 70.38 |
| 20 | 23.71 | 27.16 | 31.11 | 35.63 | 40.81 | 46.75 | 53.54 | 61.33 | 70.24 | 80.45 | 92.15 |
| 30 | 28.57 | 32.88 | 37.84 | 43.55 | 50.12 | 57.68 | 66.38 | 76.39 | 87.92 | 101.18 | 116.44 |
| 40 | 34.42 | 39.80 | 46.02 | 53.22 | 61.54 | 71.16 | 82.28 | 95.15 | 110.02 | 127.22 | 147.11 |
| 50 | 41.46 | 48.17 | 55.96 | 65.02 | 75.54 | 87.77 | 101.98 | 118.48 | 137.66 | 159.94 | 185.83 |
| 60 | 49.92 | 58.28 | 68.03 | 79.42 | 92.72 | 108.24 | 126.36 | 147.51 | 172.20 | 201.03 | 234.68 |
| 70 | 60.10 | 70.50 | 82.69 | 96.99 | 113.77 | 133.45 | 156.53 | 183.61 | 215.37 | 252.62 | 296.32 |

**Table S2a Beta stiffness index (β) values for males corresponding to specified z-scores**

|  | **z-score** | | | | | | | | | | |
| --- | --- | --- | --- | --- | --- | --- | --- | --- | --- | --- | --- |
| Age | **-2.5** | **-2** | **-1.5** | **-1** | **-0.5** | **0** | **0.5** | **1** | **1.5** | **2** | **2.5** |
| 10 | 1.42 | 1.62 | 1.86 | 2.13 | 2.45 | 2.80 | 3.21 | 3.69 | 4.22 | 4.84 | 5.55 |
| 20 | 1.69 | 1.95 | 2.26 | 2.61 | 3.02 | 3.49 | 4.03 | 4.66 | 5.39 | 6.23 | 7.20 |
| 30 | 1.98 | 2.31 | 2.69 | 3.14 | 3.66 | 4.27 | 4.98 | 5.81 | 6.77 | 7.90 | 9.21 |
| 40 | 2.29 | 2.69 | 3.16 | 3.72 | 4.37 | 5.14 | 6.05 | 7.12 | 8.37 | 9.84 | 11.58 |
| 50 | 2.59 | 3.08 | 3.65 | 4.33 | 5.14 | 6.09 | 7.23 | 8.57 | 10.17 | 12.06 | 14.30 |
| 60 | 2.89 | 3.46 | 4.14 | 4.95 | 5.93 | 7.09 | 8.48 | 10.15 | 12.14 | 14.52 | 17.37 |
| 70 | 3.17 | 3.83 | 4.62 | 5.57 | 6.73 | 8.11 | 9.79 | 11.81 | 14.25 | 17.19 | 20.75 |

**Table S2b Beta stiffness index (β) values for females corresponding to specified z-scores**

|  | **z-score** | | | | | | | | | | |
| --- | --- | --- | --- | --- | --- | --- | --- | --- | --- | --- | --- |
| Age | **-2.5** | **-2** | **-1.5** | **-1** | **-0.5** | **0** | **0.5** | **1** | **1.5** | **2** | **2.5** |
| 10 | 1.86 | 2.08 | 2.33 | 2.61 | 2.92 | 3.27 | 3.66 | 4.10 | 4.59 | 5.14 | 5.75 |
| 20 | 2.19 | 2.47 | 2.80 | 3.16 | 3.57 | 4.04 | 4.57 | 5.17 | 5.84 | 6.61 | 7.47 |
| 30 | 2.47 | 2.82 | 3.22 | 3.68 | 4.20 | 4.80 | 5.48 | 6.26 | 7.15 | 8.17 | 9.33 |
| 40 | 2.81 | 3.25 | 3.74 | 4.32 | 4.98 | 5.75 | 6.63 | 7.65 | 8.82 | 10.18 | 11.74 |
| 50 | 3.23 | 3.76 | 4.38 | 5.11 | 5.95 | 6.94 | 8.08 | 9.42 | 10.97 | 12.79 | 14.90 |
| 60 | 3.73 | 4.40 | 5.17 | 6.09 | 7.17 | 8.43 | 9.93 | 11.68 | 13.75 | 16.18 | 19.05 |
| 70 | 4.35 | 5.17 | 6.15 | 7.31 | 8.69 | 10.33 | 12.29 | 14.61 | 17.36 | 20.64 | 24.54 |

**Table S3a Arterial compliance (AC; mm^2^/kPa) values for males corresponding to specified z-scores**

|  | **z-score** | | | | | | | | | | |
| --- | --- | --- | --- | --- | --- | --- | --- | --- | --- | --- | --- |
| Age | **-2.5** | **-2** | **-1.5** | **-1** | **-0.5** | **0** | **0.5** | **1** | **1.5** | **2** | **2.5** |
| 10 | 0.77 | 0.89 | 1.03 | 1.19 | 1.37 | 1.56 | 1.77 | 2.01 | 2.26 | 2.54 | 2.85 |
| 20 | 0.65 | 0.76 | 0.89 | 1.03 | 1.20 | 1.38 | 1.58 | 1.80 | 2.04 | 2.30 | 2.60 |
| 30 | 0.52 | 0.62 | 0.73 | 0.86 | 1.00 | 1.17 | 1.35 | 1.55 | 1.77 | 2.02 | 2.29 |
| 40 | 0.42 | 0.51 | 0.61 | 0.73 | 0.86 | 1.01 | 1.18 | 1.36 | 1.57 | 1.80 | 2.06 |
| 50 | 0.35 | 0.43 | 0.53 | 0.63 | 0.75 | 0.89 | 1.05 | 1.22 | 1.42 | 1.64 | 1.88 |
| 60 | 0.30 | 0.38 | 0.46 | 0.56 | 0.67 | 0.81 | 0.95 | 1.12 | 1.31 | 1.52 | 1.76 |
| 70 | 0.27 | 0.34 | 0.42 | 0.51 | 0.62 | 0.75 | 0.89 | 1.05 | 1.24 | 1.45 | 1.68 |

**Table S3b Arterial compliance (AC; mm^2^/kPa) values for females corresponding to specified z-scores**

|  | **z-score** | | | | | | | | | | |
| --- | --- | --- | --- | --- | --- | --- | --- | --- | --- | --- | --- |
| Age | **-2.5** | **-2** | **-1.5** | **-1** | **-0.5** | **0** | **0.5** | **1** | **1.5** | **2** | **2.5** |
| 10 | 0.74 | 0.86 | 1.00 | 1.15 | 1.32 | 1.51 | 1.71 | 1.94 | 2.19 | 2.46 | 2.75 |
| 20 | 0.60 | 0.71 | 0.82 | 0.96 | 1.11 | 1.27 | 1.45 | 1.65 | 1.88 | 2.12 | 2.39 |
| 30 | 0.45 | 0.54 | 0.64 | 0.75 | 0.87 | 1.01 | 1.17 | 1.34 | 1.53 | 1.74 | 1.98 |
| 40 | 0.36 | 0.43 | 0.52 | 0.61 | 0.72 | 0.84 | 0.98 | 1.13 | 1.30 | 1.49 | 1.70 |
| 50 | 0.30 | 0.37 | 0.44 | 0.53 | 0.62 | 0.73 | 0.86 | 1.00 | 1.15 | 1.33 | 1.52 |
| 60 | 0.27 | 0.33 | 0.40 | 0.48 | 0.57 | 0.67 | 0.79 | 0.92 | 1.07 | 1.23 | 1.42 |
| 70 | 0.26 | 0.32 | 0.38 | 0.46 | 0.55 | 0.65 | 0.77 | 0.90 | 1.04 | 1.21 | 1.39 |

**Table S4a Local pulse wave velocity values (PWV; m/s) for males corresponding to specified z-scores**

|  | **z-score** | | | | | | | | | | |
| --- | --- | --- | --- | --- | --- | --- | --- | --- | --- | --- | --- |
| Age | **-2.5** | **-2** | **-1.5** | **-1** | **-0.5** | **0** | **0.5** | **1** | **1.5** | **2** | **2.5** |
| 10 | 2.81 | 3.00 | 3.21 | 3.42 | 3.66 | 3.91 | 4.17 | 4.45 | 4.76 | 5.08 | 5.43 |
| 20 | 2.94 | 3.16 | 3.40 | 3.65 | 3.92 | 4.21 | 4.52 | 4.86 | 5.22 | 5.61 | 6.02 |
| 30 | 3.24 | 3.50 | 3.78 | 4.09 | 4.41 | 4.77 | 5.15 | 5.57 | 6.01 | 6.50 | 7.02 |
| 40 | 3.51 | 3.82 | 4.15 | 4.51 | 4.90 | 5.32 | 5.79 | 6.29 | 6.83 | 7.42 | 8.07 |
| 50 | 3.76 | 4.10 | 4.49 | 4.90 | 5.36 | 5.86 | 6.40 | 7.00 | 7.65 | 8.36 | 9.13 |
| 60 | 3.96 | 4.35 | 4.78 | 5.25 | 5.78 | 6.35 | 6.98 | 7.67 | 8.43 | 9.27 | 10.19 |
| 70 | 4.11 | 4.54 | 5.02 | 5.55 | 6.14 | 6.78 | 7.50 | 8.29 | 9.17 | 10.14 | 11.21 |

**Table S4b Local pulse wave velocity (PWV; m/s) values for females corresponding to specified z-scores**

|  | **z-score** | | | | | | | | | | |
| --- | --- | --- | --- | --- | --- | --- | --- | --- | --- | --- | --- |
| Age | **-2.5** | **-2** | **-1.5** | **-1** | **-0.5** | **0** | **0.5** | **1** | **1.5** | **2** | **2.5** |
| 10 | 2.64 | 2.82 | 3.01 | 3.22 | 3.44 | 3.67 | 3.92 | 4.18 | 4.46 | 4.77 | 5.09 |
| 20 | 2.91 | 3.11 | 3.34 | 3.57 | 3.83 | 4.10 | 4.39 | 4.70 | 5.03 | 5.39 | 5.78 |
| 30 | 3.21 | 3.45 | 3.70 | 3.98 | 4.28 | 4.59 | 4.94 | 5.30 | 5.70 | 6.12 | 6.58 |
| 40 | 3.53 | 3.80 | 4.10 | 4.42 | 4.76 | 5.13 | 5.53 | 5.96 | 6.42 | 6.92 | 7.46 |
| 50 | 3.86 | 4.17 | 4.51 | 4.88 | 5.27 | 5.70 | 6.16 | 6.66 | 7.20 | 7.79 | 8.42 |
| 60 | 4.20 | 4.56 | 4.94 | 5.36 | 5.82 | 6.31 | 6.84 | 7.42 | 8.05 | 8.73 | 9.46 |
| 70 | 4.56 | 4.96 | 5.40 | 5.87 | 6.39 | 6.95 | 7.56 | 8.23 | 8.95 | 9.74 | 10.59 |

These models have also been used to construct a simple calculator which is available as an Excel spreadsheet that can be downloaded separately. The layout of the spreadsheet, with sample data, is shown here:

**ETIC Calculator of Z Values**

| Enter the value of the appropriate parameter below, together with age and gender. | | | | | | | | | | | | |  |
| --- | --- | --- | --- | --- | --- | --- | --- | --- | --- | --- | --- | --- | --- |
| The Z-score will be calculated automatically. | | | | | | | | |  |  |  |  |  |
| A Z-score of 0 is average. | | | |  |  |  |  |  |  |  |  |  |  |
| Z-scores less than -2 or greater than 2 are fairly unusual, representing respectively the bottom  and top 2.5% of the population. | | | | | | | | | | | | | |
|  | | |  |  | | | |  |  |  | |  |  |
| Epsilon value | Age | Gender (0=male, 1=female) | | | | | z-score | | | |  |  |  |
|  |  |  | | |  |  |  | | | |  |  |  |
| 75 | 40 | 0 | | |  |  | 0.004081 | | | |  |  |  |
|  |  |  | | |  |  |  | | | |  |  |  |
| β value | Age | Gender (0=male, 1=female) | | | | | z-score | | | |  |  |  |
|  |  |  | | |  |  |  | | | |  |  |  |
| 5.7 | 40 | 0 | | |  |  | 0.004132 | | | |  |  |  |
|  |  |  | | |  |  |  | | | |  |  |  |
| PWV value | Age | Gender (0=male, 1=female) | | | | | z-score | | | |  |  |  |
|  |  |  | | |  |  |  | | | |  |  |  |
| 5.2 | 40 | 0 | | |  |  | -0.04126 | | | |  |  |  |
|  |  |  | | |  |  |  | | | |  |  |  |
| AC value | Age | Gender (0=male, 1=female) | | | | | z-score | | | |  |  |  |
|  |  |  | | |  |  |  | | | |  |  |  |
| 1 | 40 | 0 | | |  |  | -0.02717 | | | |  |  |  |
