# Supplementary figures and images for: Age-specific reference values for carotid arterial stiffness estimated by ultrasonic wall tracking
Source: J Hum Hypertens. 2019 Aug 21;34(3):214–22. doi: 10.1038/s41371-019-0228-5 (PMC8076029; doi:10.1038/s41371-019-0228-5)

## Slide 1
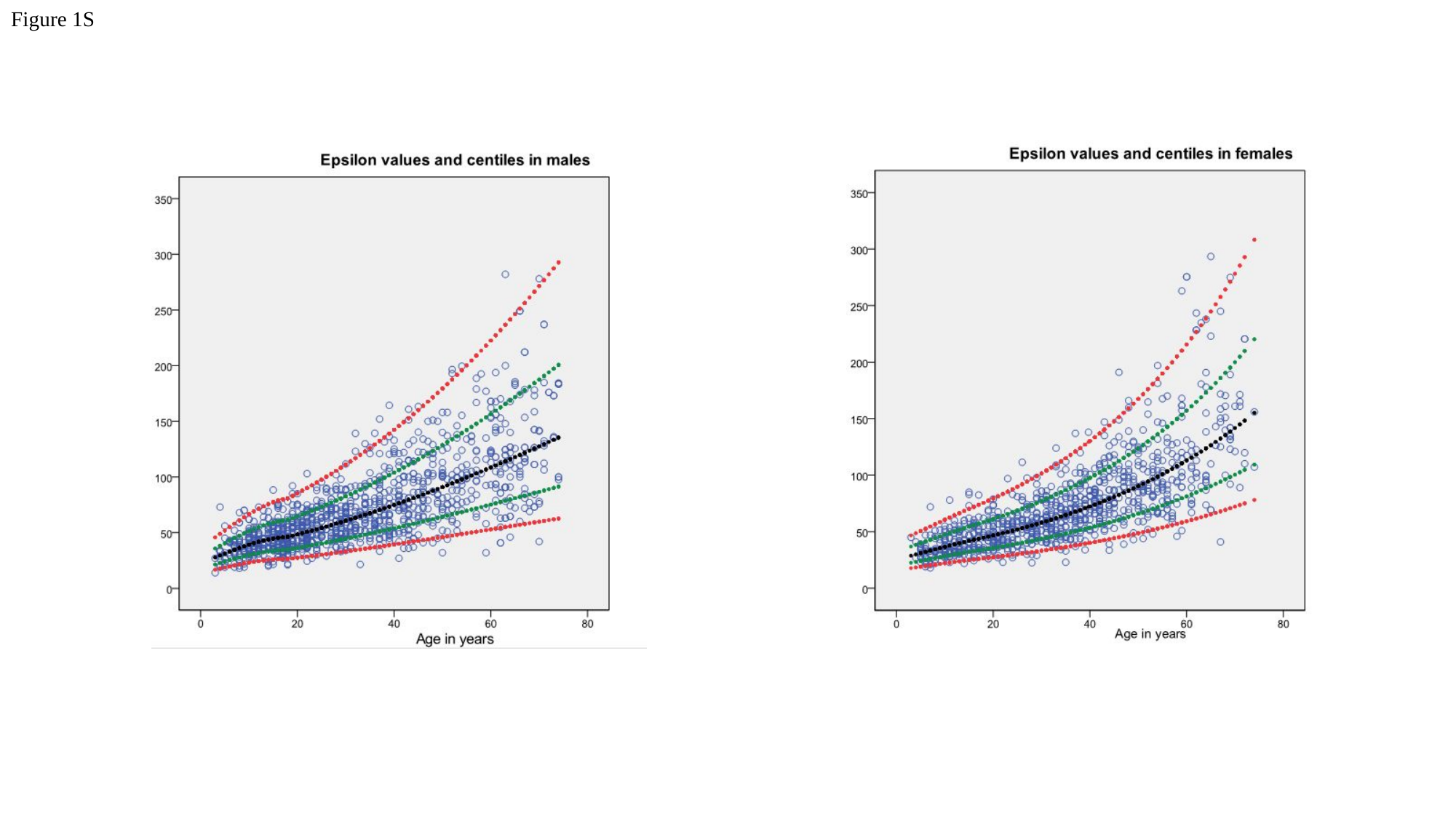

Figure 1S

Supplement: Supplementary file 2 — Supplemental Figure [file 41371_2019_228_MOESM2_ESM.pptx]
